# Review of Galectin-3 and the Collagen Marker PIIINP in Myocardial Fibrosis in Dilated Cardiomyopathy: Predictive Value for Major Adverse Cardiovascular Events

**DOI:** 10.31083/RCM52258

**Published:** 2026-10-21

**Authors:** Xiduo Zhao, Lianyu Hua, Youjun Xiong, Fuyou Shi, Yilin Zhong, Shangwei Zhang, Chengnan Tian

**Affiliations:** ^1^The First Clinical College, Gannan Medical University, 341000 Ganzhou, Jiangxi, China; ^2^Department of Cardiovascular Surgery, The First Affiliated Hospital of Gannan Medical University, 341000 Ganzhou, Jiangxi, China

**Keywords:** myocardial fibrosis, risk assessment, biomarkers, cardiovascular diseases, dilated cardiomyopathy, Galectin-3, type III procollagen N-terminal propeptide

## Abstract

Dilated cardiomyopathy (DCM) is characterized by ventricular dilation and systolic dysfunction, and the associated pathophysiology is closely linked to myocardial fibrosis, a key contributor to disease progression and poor prognosis. Galectin-3 (Gal-3) plays a crucial role in initiating early proinflammatory and profibrotic signaling. Meanwhile, type III procollagen N-terminal propeptide (PIIINP) serves as a marker of type III collagen synthesis and acts as an active profibrotic effector. The counterpart to PIIINP, type III procollagen C-terminal propeptide (PIIICP), is released during type III collagen maturation, reflecting distinct processing and clearance dynamics. PIIINP promotes fibrosis through integrin α2β1/discoidin domain receptor 1 (DDR1)-mediated extracellular signal-regulated kinase (ERK)/phosphatidylinositol 3-kinase/protein kinase B (PI3K/Akt) signaling and contributes to the establishment of a Gal-3–transforming growth factor-β (TGF-β)–PIIINP positive feedback loop, thereby linking inflammation to extracellular matrix deposition. Recently, Gal-3 and PIIINP have gained recognition as valuable biomarkers for the diagnosis, risk stratification, and prognosis assessment of DCM. Unlike previous reviews that focused solely on individual fibrosis markers or static histological assessments, this review uniquely highlights the complementary roles of Gal-3 and PIIINP. These markers are crucial for understanding inflammatory-fibrotic activation and dynamic collagen turnover. This review systematically outlines the expression patterns of these markers across various stages of DCM and underscores the additional prognostic value of the associated combined detection beyond traditional indicators. Among the components of major adverse cardiovascular events (MACE), heart failure hospitalization and cardiovascular mortality are most strongly associated with elevated levels of Gal-3 and PIIINP in patients with DCM. Specifically, Gal-3 levels of ≥14.54 ng/mL are closely linked to increased risks of heart failure readmission and sudden cardiac death. In contrast, elevated PIIINP levels are most strongly associated with heart failure hospitalization and ventricular arrhythmias. This review aims to elucidate the specific biological functions of Gal-3 and PIIINP, address both mechanistic and clinical uncertainties, and summarize evidence-based thresholds for risk stratification. This review also seeks to provide theoretical insights and clinical guidance for early intervention and personalized anti-fibrotic therapy in DCM. A systematic search was conducted in PubMed, Embase, and Web of Science, covering literature from inception to March 2026. The search used keywords such as “dilated cardiomyopathy”, “myocardial fibrosis”, “Galectin-3”, “PIIINP”, “biomarker”, and “cardiovascular events”. The review included studies comprising clinical trials, observational research, and mechanistic investigations of Gal-3 and PIIINP in DCM. Reviews, case reports, and non-English literature were excluded. Data were synthesized using a narrative review approach.

## 1. Introduction

Dilated cardiomyopathy (DCM) is a primary heart muscle disease characterized by ventricular enlargement and impaired systolic function, with myocardial fibrosis at its core. This fibrosis leads to irreversible structural and functional changes in the heart, increasing the risk of heart failure and major adverse cardiovascular events (MACE) [1]. Recent advances in molecular imaging and biomarker research have identified Galectin-3 (Gal-3) and type III procollagen N-terminal propeptide (PIIINP) as key regulators and indicators of myocardial fibrosis activity. These biomarkers are now central to DCM risk assessment and the development of therapeutic targets. This review takes a narrative approach, selecting studies based on their relevance to Gal-3, PIIINP, and myocardial fibrosis in DCM. Preference was given to clinical observational studies, mechanistic investigations, and meta-analyses published in English, while excluding case reports, reviews, and non-clinical studies.

While individual studies have explored the links between Gal-3 or PIIINP and myocardial fibrosis in DCM, most reviews tend to focus on a single biomarker or isolated pathological aspects [2]. There is a notable absence of comprehensive analyses that examine the complementary roles of Gal-3 and PIIINP and their combined predictive value for MACE. This review aims to fill this gap by systematically exploring their molecular mechanisms, expression patterns, and prognostic utility beyond traditional markers. The goal is to offer new insights for risk stratification and personalized antifibrotic therapy in DCM. Gal-3 and PIIINP represent two distinct yet complementary aspects of myocardial fibrosis. Gal-3 is primarily associated with systemic inflammation and the activation of pro-fibrotic signaling, whereas PIIINP directly indicates ongoing collagen synthesis and extracellular matrix turnover. Therefore, assessing both biomarkers together offers a more comprehensive and accurate evaluation of myocardial fibrosis severity and prognosis.

Gal-3, a β-galactoside-binding protein, plays a vital role in myocardial inflammation and fibrosis progression. It activates the transforming growth factor-β (TGF-β)/Smad pathway, which promotes myocardial fibroblast proliferation and collagen deposition. Clinical studies indicate that circulating Gal-3 levels are closely linked to the extent of ventricular remodeling in patients with DCM. Specifically, Gal-3 concentrations of ≥14.54 ng/mL significantly predict cardiovascular mortality and the risk of heart failure hospitalization [3]. The pro-fibrotic effects of Gal-3 may be mediated through its regulation of endoplasmic reticulum stress and impaired autophagy flux. In mouse models, knocking out the Gal-3 gene reduces age-related myocardial hypertrophy and interstitial fibrosis, along with decreased matrix metalloproteinase-9 (MMP-9) expression. However, some studies report no significant correlation between circulating Gal-3 levels and collagen volume fraction (CVF) in endomyocardial biopsies. This suggests Gal-3 might indicate systemic inflammatory status rather than local fibrosis. This discrepancy can be explained by understanding that circulating Gal-3 primarily reflects systemic inflammation and fibrotic activation, whereas CVF represents the static histological fibrosis burden.

PIIINP, a byproduct of type III collagen synthesis, serves as a marker for myocardial fibrosis activity [4]. In contrast, the Type III procollagen C-terminal propeptide (PIIICP), cleaved from the same procollagen molecule, is more closely linked to mature collagen deposition. In patients with DCM, serum PIIINP levels have been shown to positively correlate with the extracellular volume fraction (ECV) as measured by cardiac magnetic resonance (CMR). Research indicates that combining PIIINP with late gadolinium enhancement (LGE) can improve the effectiveness of risk stratification models. A notable single-center study reported that patients testing positive for both LGE and PIIINP had a 5-year adverse event rate of up to 42%, significantly higher than other groups. However, a multicenter study found a smaller risk difference.

Current research on Gal-3 and PIIINP in DCM is primarily confined to single-biomarker analyses or small-scale observational studies. These studies often lack a comprehensive synthesis of the biomarkers’ complementary roles in inflammation-fibrosis interactions and dynamic extracellular matrix (ECM) remodeling [5]. Moreover, there is no systematic review that clearly defines their combined incremental prognostic value beyond conventional markers, nor summarizes evidence-based thresholds for guiding risk stratification and antifibrotic interventions. This review aims to systematically summarize the molecular mechanisms of Gal-3 and PIIINP in myocardial fibrosis, clarify their dynamic expression characteristics across different DCM stages, evaluate their individual and combined predictive value for MACE, and discuss current research controversies and future directions. The scope focuses on non-ischemic DCM and clinical biomarker applications.

## 2. Biological Properties of Gal-3 and Its Role in Myocardial Fibrosis

### 2.1 Molecular Structure and Function of Gal-3

Gal-3 is the sole chimeric member of the galectin family, characterized by a C-terminal carbohydrate recognition domain (CRD) and an N-terminal domain (NTD). The CRD contains a highly conserved β-galactoside binding site that specifically recognizes glycan complexes with β-galactoside structures, such as N-acetyl-D-galactosamine (LacNAc). Research indicates that the CH-π interaction of residue W181 is crucial for maintaining ligand-binding stability during Gal-3’s interaction with LacNAc [6]. This interaction, along with a network of hydrogen bonds, underpins Gal-3’s sugar recognition mechanism. The NTD, comprising about 120 amino acids with eight collagen-like tandem repeats rich in proline-glycine, exhibits intrinsic disorder. However, accelerated molecular dynamics simulations and nuclear magnetic resonance (NMR) studies have shown that the NTD forms transient contacts with the CRD. This dynamic interaction plays a significant role in regulating Gal-3 (Fig. 1).

**Fig. 1.**
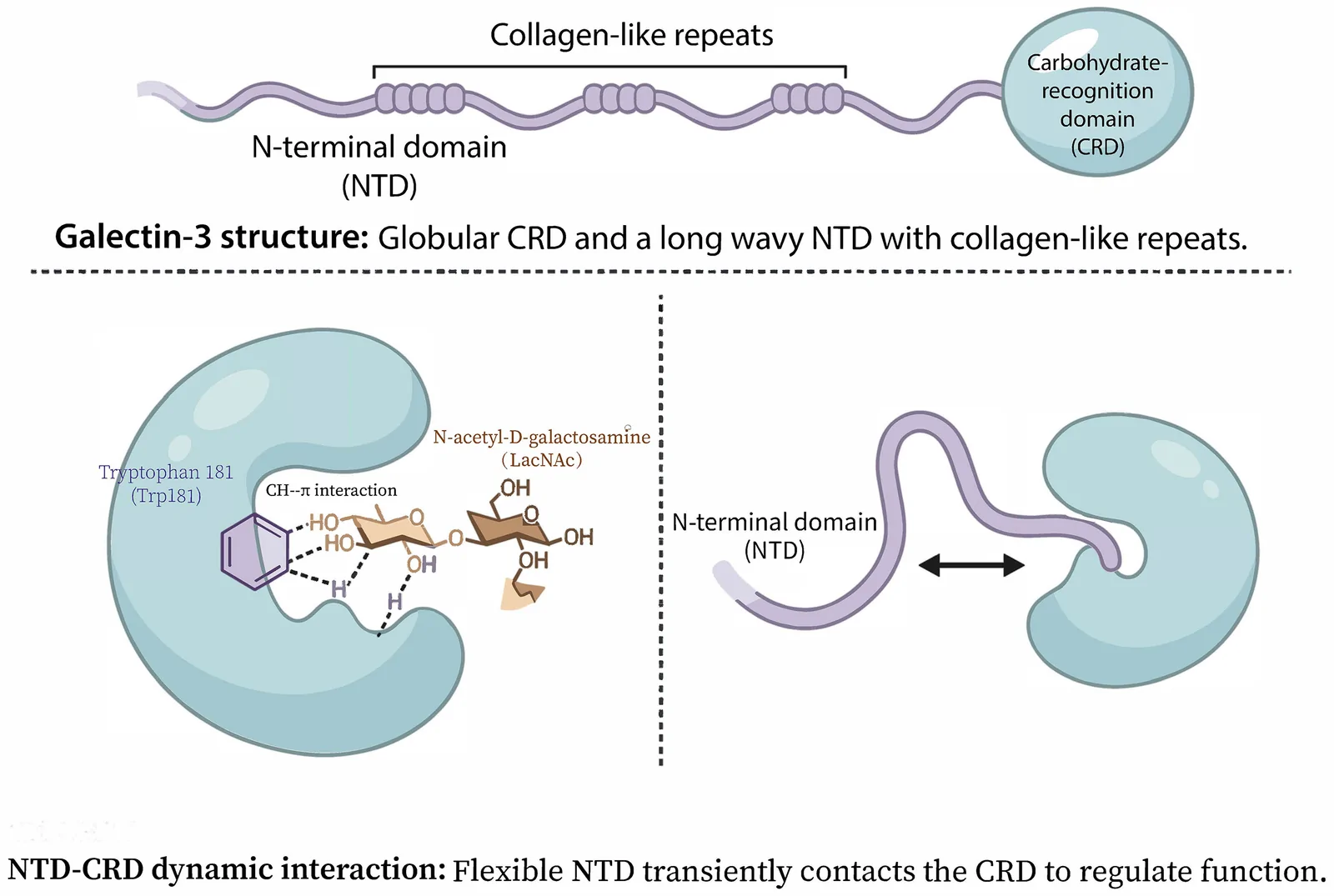
**Galectin-3 (Gal-3) consists of a C-terminal carbohydrate recognition domain (CRD) and an N-terminal domain (NTD), and the flexible NTD can transiently interact with CRD to modulate its biological function**.

Gal-3 serves as an extracellular signaling molecule, essential for cell adhesion, migration, and signal transduction via its CRD that binds glycans [7]. Studies indicate that Gal-3 forms heterodimers with the chemokine C-X-C chemokine ligand 12 (CXCL12) by interacting with CXCL12’s β-chain 1 and Gal-3’s F-face residues. This interaction is pivotal in regulating leukocyte chemotaxis and C-X-C chemokine receptor 4 (CXCR4) signaling. Inside the cell, Gal-3 plays a role in membrane receptor endocytosis and retrograde transport through NTD-mediated oligomerization, which relies on its ability to recognize specific N-glycan patterns.

The expression and secretion of Gal-3 are influenced by several factors [8]. Changes in nutritional status dynamically affect its secretion through O-linked N-acetylglucosamine glycosylation (O-GlcNAc), a post-translational modification that varies significantly between the cytoplasmic and secreted forms of Gal-3. Interestingly, Gal-3 acts as a double-edged sword: it provides protective effects during acute injuries but can drive pathological progression in chronic inflammatory conditions. This functional diversity is closely linked to its molecular structural plasticity, glycan-binding specificity, and cellular localization, making it a potential therapeutic target for various diseases.

### 2.2 Molecular Mechanisms of Gal-3 in Myocardial Fibrosis

Gal-3, a β-galactoside-binding lectin, plays a vital role in the development and progression of myocardial fibrosis. It mainly promotes collagen deposition by activating fibroblasts and regulating ECM components [9]. Furthermore, Gal-3 interacts with TGF-β receptors, stabilizing the receptor complex to enhance downstream Smad2/3 phosphorylation. This interaction facilitates fibroblast-to-myofibroblast transdifferentiation and increases collagen gene expression. Preclinical studies consistently demonstrate that inhibiting Gal-3 reduces fibrosis in myocardial tissue (Fig. 2).

**Fig. 2.**
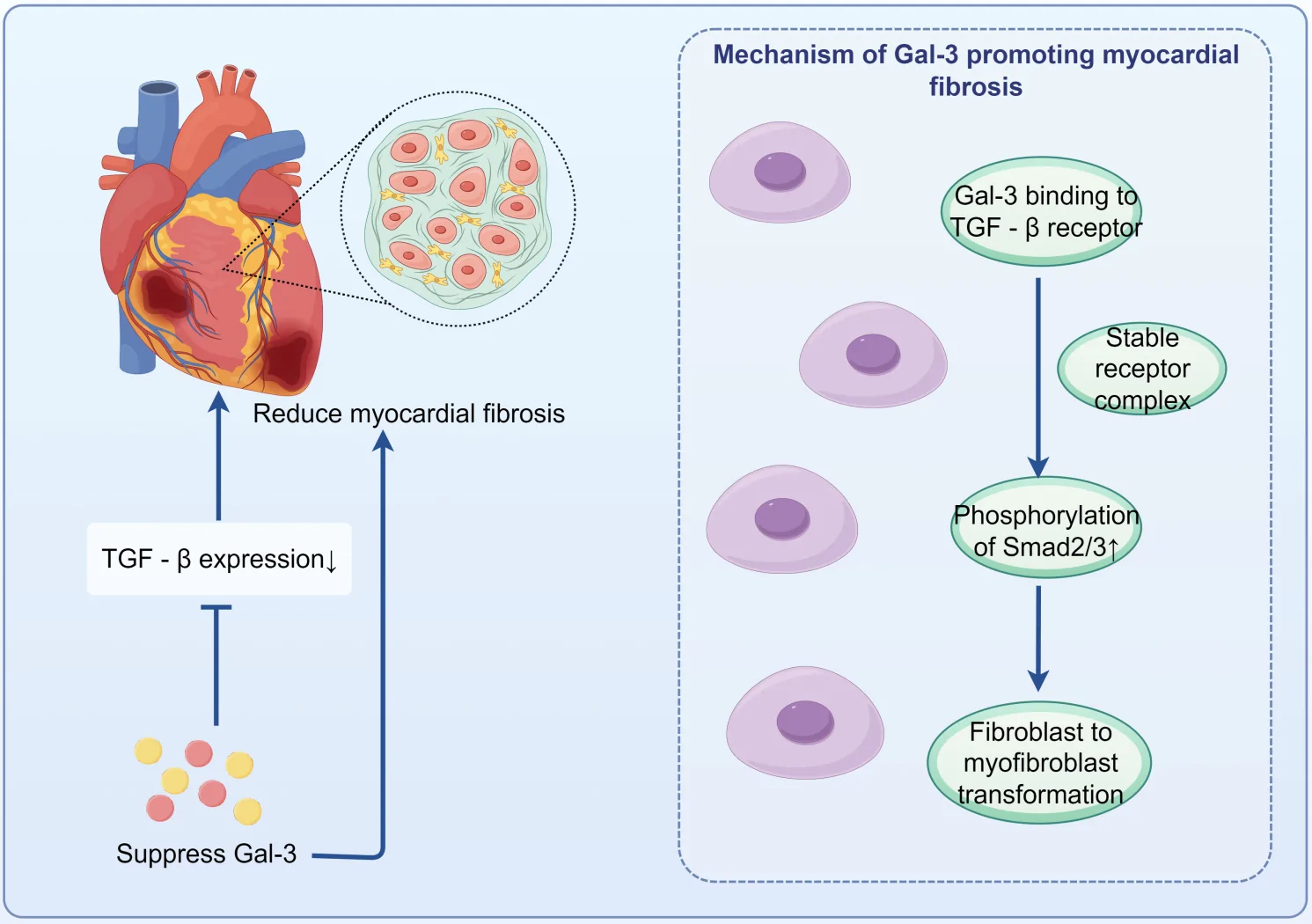
**Mechanism by which Galectin-3 (Gal-3) promotes myocardial fibrosis: Gal-3 binds to the transforming growth factor-β (TGF-β) receptor and stabilizes the receptor complex, thereby enhancing phosphorylation of downstream Smad2/3 and driving fibroblast-to-myofibroblast transformation**. In the context of myocardial fibrosis, inhibition of Galectin-3 reduces TGF-β expression, which attenuates fibrosis and improves cardiac function.

In mouse models, knocking out the Gal-3 gene alleviated myocardial fibrosis and decreased the expression of TGF-β and MMP-9. This suggests that Gal-3 promotes fibrosis progression through the regulation of the TGF-β signaling pathway [10]. Additionally, Gal-3 worsens myocardial remodeling by inhibiting the expression of Sirtuin 1 (SIRT1) and Sirtuin 7 (SIRT7) [11]. Gal-3 is pivotal in myocardial fibrosis by modulating inflammatory responses. It promotes interactions between macrophages and fibroblasts, creating a pro-fibrotic microenvironment [12]. Through the Toll-like receptor 4 (TLR4)/myeloid differentiation primary response protein 88 (MyD88)/nuclear factor-kappa B (NF-κB) signaling pathway, Gal-3 boosts the release of inflammatory cytokines, including interleukin-1β (IL-1β), interleukin-18 (IL-18), and tumor necrosis factor-α (TNF-α), which exacerbate myocardial injury and fibrosis. Moreover, Gal-3 promotes M2 macrophage polarization by upregulating arginase-1 (Arg-1) and inducible nitric oxide synthase (iNOS), further intensifying the fibrotic response [11].

### 2.3 Expression Dynamics of Gal-3 in Dilated Cardiomyopathy

Gal-3 expression in DCM varies significantly with the disease’s progression [13]. In patients newly diagnosed with DCM, serum Gal-3 levels are markedly elevated but tend to decrease as the disease progresses. Research shows that baseline Gal-3 levels in patients with a disease duration of ≤6 months are significantly higher than those observed at 3-month and 12-month follow-ups. This pattern suggests that Gal-3 plays a crucial role in the early phase of myocardial fibrosis by activating macrophages and promoting myofibroblast conversion. In a mouse model of DCM, Gal-3 expression was significantly upregulated during the early phase of myocardial remodeling, triggered by cardiac-specific Hippo pathway activation. This upregulation was notably absent in Gal-3 knockout mice.

Unlike the acute phase, Gal-3 levels in chronic DCM patients remain relatively stable. Research indicates that in patients with chronic DCM lasting over six months, Gal-3 levels at baseline, 3-month, and 12-month follow-ups were 13.82 ± 3.05 ng/mL, 13.75 ± 2.91 ng/mL, and 13.68 ± 3.12 ng/mL, respectively, showing no statistically significant differences. This stability suggests a slower progression of fibrosis during the chronic phase. Notably, Gal-3 levels in the chronic phase remain significantly higher than those in healthy controls, highlighting its continued role in myocardial remodeling [11]. Furthermore, CMR studies in non-ischemic DCM patients have shown a positive correlation between Gal-3 levels and the extent of replacement fibrosis detected by LGE.

Gal-3 undergoes dynamic changes that correspond to its dual role in myocardial fibrosis. In the acute phase, it promotes fibrosis by increasing inflammatory cell infiltration and collagen deposition. In contrast, during the chronic phase, Gal-3 primarily maintains the established fibrosis. This unique expression pattern makes it a potential biomarker for tracking the progression of DCM and predicting MACE. Importantly, Gal-3 indicates fibrosis activity rather than the static fibrosis burden. It provide crucial insights into the biological role of Gal-3 at different stages of DCM.

### 2.4 Relationship Between Gal-3 and Other Fibrosis-Related Markers

Gal-3 is a key regulator in fibrosis, interacting with markers involved in ECM remodeling to create a complex network [14]. In DCM-specific cohorts, circulating Gal-3 levels positively correlate with osteopontin (OPN), tissue inhibitor of metalloproteinase-1 (TIMP-1), matrix metalloproteinase-2 (MMP-2), and PIIICP. This indicates Gal-3’s comprehensive role in ECM homeostasis. Studies reveal significant correlations between serum Gal-3 and OPN, TIMP-1, PIIICP, and MMP-2, suggesting elevated Gal-3 is linked to increased collagen synthesis, inhibited matrix degradation, and activation of pro-fibrotic cytokines. This broad association supports the idea that Gal-3 serves as a “central coordinator” of ECM remodeling. It may promote myocardial fibrosis progression by activating macrophages and fibroblasts, thereby upregulating other pro-fibrotic mediators [15].

Research extrapolated from non-DCM conditions supports the association between Gal-3 and fibrosis. In cases of renal fibrosis, urinary Gal-3 levels positively correlate with PIIINP [16]. Similarly, in heart failure patients, both baseline and 12-month urinary Gal-3 levels show a correlation with urinary PIIINP, reinforcing the strong connection between Gal-3 and type III collagen synthesis. Furthermore, a study on hypertrophic cardiomyopathy (HCM) identified a link between Gal-3 and extracellular volume fraction (ECV), although not all studies concur. This suggests Gal-3’s unique potential in identifying specific types of myocardial fibrosis [17].

Circulating Gal-3 does not correlate with the histological fibrosis burden, such as CVF, but rather reflects ongoing fibrotic activity. In patients with DCM, Gal-3 levels in the blood do not directly correlate with fibrosis severity as assessed by endomyocardial biopsy. Similarly, a systematic review of chronic Chagas cardiomyopathy (CCC) found insufficient evidence to link Gal-3 expression with myocardial fibrosis. These findings suggest that Gal-3 may indicate active fibrotic processes rather than established fibrotic scarring. Therefore, combining Gal-3 with dynamic markers like PIIINP, which directly reflect collagen synthesis and degradation, offers a comprehensive assessment of fibrotic activity and disease progression risk [18]. This combination provides a more precise opportunity for clinical intervention.

## 3. Role of Collagen Marker PIIINP in Myocardial Fibrosis

### 3.1 Biological Significance and Detection Methods of PIIINP

PIIINP is a byproduct released during the synthesis of type III collagen. While PIIINP reflects collagen synthesis activity related to myocardial fibrosis, it is not exclusively a biomarker for this condition [17,19]. In the development of myocardial fibrosis, type III collagen, an early extracellular matrix component, shows increased synthesis, which is crucial for tissue repair and fibrotic remodeling. The release of PIIINP aligns with collagen fiber formation, allowing its concentration changes to partially indicate the severity of myocardial fibrosis, though it is limited by non-cardiac sources [20]. Serum PIIINP levels are notably higher in patients with DCM and post-myocardial infarction ventricular remodeling, showing a positive correlation with the extent of myocardial fibrosis [21,22]. Furthermore, elevated PIIINP levels are linked to left ventricular diastolic dysfunction and a higher risk of adverse cardiovascular events, highlighting its potential predictive value in disease progression despite its systemic origin.

Detection of PIIINP primarily relies on serological methods, with enzyme-linked immunosorbent assay (ELISA) and automated immunoassays being the most commonly used techniques. These methods provide high sensitivity and specificity, enabling precise quantification of PIIINP concentrations. The reference range for adults is 4.42–16.0 µg/L and remains consistent across genders. Recently, the microfluidic light-induced chemiluminescence assay (LICA) has enhanced detection efficiency, allowing for rapid quantitative analysis of PIIINP in multiplex testing, thus facilitating the noninvasive diagnosis of myocardial fibrosis. However, there is significant measurement variability across different assay platforms. This variability severely affects result reproducibility and clinical applicability. It hinders cross-study comparability, complicates the establishment of unified diagnostic cutoff values, and limits the reliability of longitudinal monitoring for disease progression or treatment response.

Monitoring PIIINP levels dynamically is essential for evaluating the effectiveness of anti-fibrotic therapies. For instance, spironolactone treatment has been demonstrated to lower PIIINP levels and enhance left ventricular diastolic function [23]. Furthermore, combining PIIINP measurements with LGE on CMR significantly improves prognostic predictions in patients with DCM. However, non-cardiac factors such as liver fibrosis, obesity, and aging can influence PIIINP levels. Thus, integrating assessments with Gal-3 is crucial for accurate evaluations.

### 3.2 Molecular Mechanisms of PIIINP in Myocardial Fibrosis

PIIINP, encoded by the *COL3A1* gene, is produced by cardiac fibroblasts, myofibroblasts, and activated smooth muscle cells after myocardial injury. During collagen synthesis, type III procollagen is formed inside cells with both N-terminal and C-terminal propeptides [24]. Once secreted into the extracellular space, specific procollagen N-proteinases cleave PIIINP and PIIICP from the mature collagen triple helix. PIIINP is not integrated into collagen fibrils but is released into the bloodstream, where it remains stable and measurable. This makes it a direct indicator of *COL3A1* transcription and ECM synthesis [25].

PIIINP is more than a passive marker; it actively contributes to myocardial fibrosis by interacting with integrin α2β1 and discoidin domain receptor 1 (DDR1) on cardiac fibroblasts. This interaction triggers the extracellular signal-regulated kinase (ERK) and phosphatidylinositol 3-kinase/protein kinase B (PI3K/Akt) pathways, which promote fibroblast proliferation, migration, and myofibroblast differentiation [26]. Consequently, there is an upregulation of *COL3A1* and type I collagen (*COL1A1*) expression. PIIINP also increases connective tissue growth factor (CTGF), further enhancing ECM deposition. Additionally, PIIINP inhibits matrix metalloproteinase (MMP) activity while boosting tissue inhibitor of metalloproteinase (TIMP) expression, leading to ECM accumulation. Moreover, PIIINP functions as a downstream effector in Gal-3-driven inflammation. Gal-3 activates the TGF-β/Smad pathway, which increases *COL3A1* transcription and PIIINP release. In turn, PIIINP enhances Gal-3 expression through a positive feedback loop, perpetuating pro-fibrotic signaling. This Gal-3→TGF-β→PIIINP axis connects upstream inflammation with downstream ECM synthesis, establishing a central fibrotic cascade in DCM. Unlike Gal-3, PIIINP does not directly regulate inflammation but intensifies fibrosis initiated by inflammatory mediators.

### 3.3 Clinical Research Advances on PIIINP in Dilated Cardiomyopathy

Serum levels of PIIINP are significantly elevated in patients with DCM and are closely associated with cardiac structural remodeling and functional impairment. However, PIIINP is not specific to the heart, as it reflects systemic collagen turnover affected by fibrosis in the liver, skin, and musculoskeletal system. In individuals with non-ischemic dilated cardiomyopathy (NIDCM), PIIINP levels are particularly higher in those showing LGE. LGE on CMR is a well-established non-invasive method for detecting myocardial replacement fibrosis, although histological assessment remains the definitive standard. Therefore, while elevated PIIINP levels indicate increased collagen synthesis linked to myocardial fibrosis, they are not cardiac-specific. These findings suggest that serum PIIINP levels are a useful, albeit non-specific, biomarker for assessing myocardial fibrosis activity in DCM patients, with its elevation closely correlating with fibrotic scarring observed on cardiac MRI [2].

PIIINP, a marker of fibrosis indicative of collagen synthesis, shows potential for monitoring the progression of DCM and evaluating treatment responses. Although PIIINP, when adjusted for known clinical predictors, did not independently predict the composite endpoint of death, heart transplantation, heart failure hospitalization, or life-threatening arrhythmias, it remains significant in assessing disease dynamics. A study involving 70 DCM patients over 12 months systematically measured various fibrosis markers, including PIIINP, at baseline, 3 months, and 12 months. This longitudinal approach allows for observing changes in collagen metabolism as the disease progresses. While the study primarily focused on Gal-3, its methodology suggests that ongoing monitoring of collagen markers like PIIINP could be crucial in assessing the effectiveness of anti-fibrotic therapies. Changes in PIIINP levels before and after treatment might indirectly indicate whether myocardial collagen synthesis is being suppressed, thus informing therapeutic adjustments to slow the progression of myocardial fibrosis. Consequently, further investigation into the clinical utility of PIIINP as a monitor for disease activity and treatment response is warranted [27].

## 4. Clinical Correlation Between Gal-3, PIIINP, and Myocardial Fibrosis

### 4.1 Correlation Analysis Between Circulating Gal-3 and Myocardial Tissue Fibrosis

Circulating Gal-3 levels do not directly correlate with the severity of myocardial fibrosis. Instead, they may indicate inflammation related to fibrosis and the metabolic status of the ECM. Importantly, Gal-3 reflects active fibrotic and inflammatory signaling rather than static histological fibrosis load. In individuals with DCM, serum Gal-3 levels correlate with PIIICP and MMP-2. However, no significant association exists between Gal-3 levels and fibrosis severity as determined by myocardial biopsy. Similarly, in patients with CCC, no direct link has been identified between Gal-3 expression and myocardial fibrosis. This lack of direct correlation may be due to Gal-3’s role as a paracrine mediator of inflammation and fibrosis activation, where its circulating levels reflect systemic pathological processes rather than localized fibrosis. In patients with acute heart failure (AHF), elevated Gal-3 levels correlate with renal tubular and myocardial injury but do not directly quantify the severity of myocardial fibrosis.

Dynamic changes in Gal-3 correspond with biomarkers of active fibrosis, highlighting its regulatory role in the fibrotic process. In ischemic cardiomyopathy, elevated Gal-3 levels worsen myocardial fibrosis by inducing endoplasmic reticulum stress. However, the Gal-3 inhibitor, modified citrus pectin, can reverse this effect. In patients with DCM, a baseline Gal-3 level of ≥14.54 ng/mL independently predicts major adverse cardiovascular events, although it is not directly linked to myocardial fibrosis. CMR shows that higher Gal-3 levels are associated with diffuse fibrosis, and this combination predicts poorer clinical outcomes in patients with focal scarring (LGE-positive) [28]. These findings indicate that Gal-3 may act as a composite biomarker for predicting disease progression by integrating multiple pathological processes, such as inflammation, ECM, and fibrotic activity [29].

### 4.2 Advantages of Combined Assessment for Fibrosis

In patients with DCM, the combined detection of Gal-3 and PIIINP enhances the assessment of myocardial fibrosis, improving diagnostic and monitoring precision [2]. Specifically, Gal-3 is involved in mediating inflammatory responses and fibrotic activation, while PIIINP directly indicates type III collagen synthesis. In contrast, type I procollagen C-terminal propeptide (PICP) is associated with type I collagen synthesis. Mechanistically, Gal-3 functions as a pro-inflammatory and pro-fibrotic trigger, activating pathways such as TGF-β/Smad and TLR4/MyD88/NF-κB to initiate fibroblast activation and collagen gene transcription [30]. PIIINP, on the other hand, acts downstream as both a marker of synthesis and a signaling effector, reflecting and amplifying collagen production. This upstream-downstream synergy allows Gal-3 to capture the initiation of the inflammatory-fibrotic process, while PIIINP measures the actual collagen synthesis activity [18]. Together, they offer a comprehensive view of fibrosis progression, which cannot be achieved by either marker alone.

In patients with non-ischemic DCM, biomarkers such as Gal-3 and PICP show a strong correlation with the extent of LGE. Their combined detection enhances risk stratification. Although LGE serves as an independent prognostic predictor for these patients, Gal-3 and PICP are closely linked to heart failure severity and provide additional predictive value for severe impairment of left ventricular ejection fraction. LGE, observed through CMR, is a well-established non-invasive reference for assessing myocardial fibrosis, with histology as the definitive standard. Specifically, elevated levels of Gal-3, PICP, and PIIINP are significantly associated with LGE-positive patients and correlate directly with LGE mass. The combined use of these biomarkers offers predictive advantages beyond individual indicators. Research suggests that Gal-3 interacts with PIIINP during the fibrotic process in DCM. As a pro-fibrotic factor, Gal-3 may enhance PIIINP release by activating the TGF-β1/Smad signaling pathway, which stimulates cardiac fibroblasts to produce more type III collagen [31]. Supporting data from other conditions show that in patients with metabolic syndrome and atrial fibrillation, Gal-3 and PIIINP levels are linked to left atrial fibrosis. Additionally, in ST-segment elevation acute coronary syndrome (STE-ACS), colchicine reduces PIIINP and modulates Gal-3. These findings, while promising, require further validation in the context of DCM (Fig. 3).

**Fig. 3.**
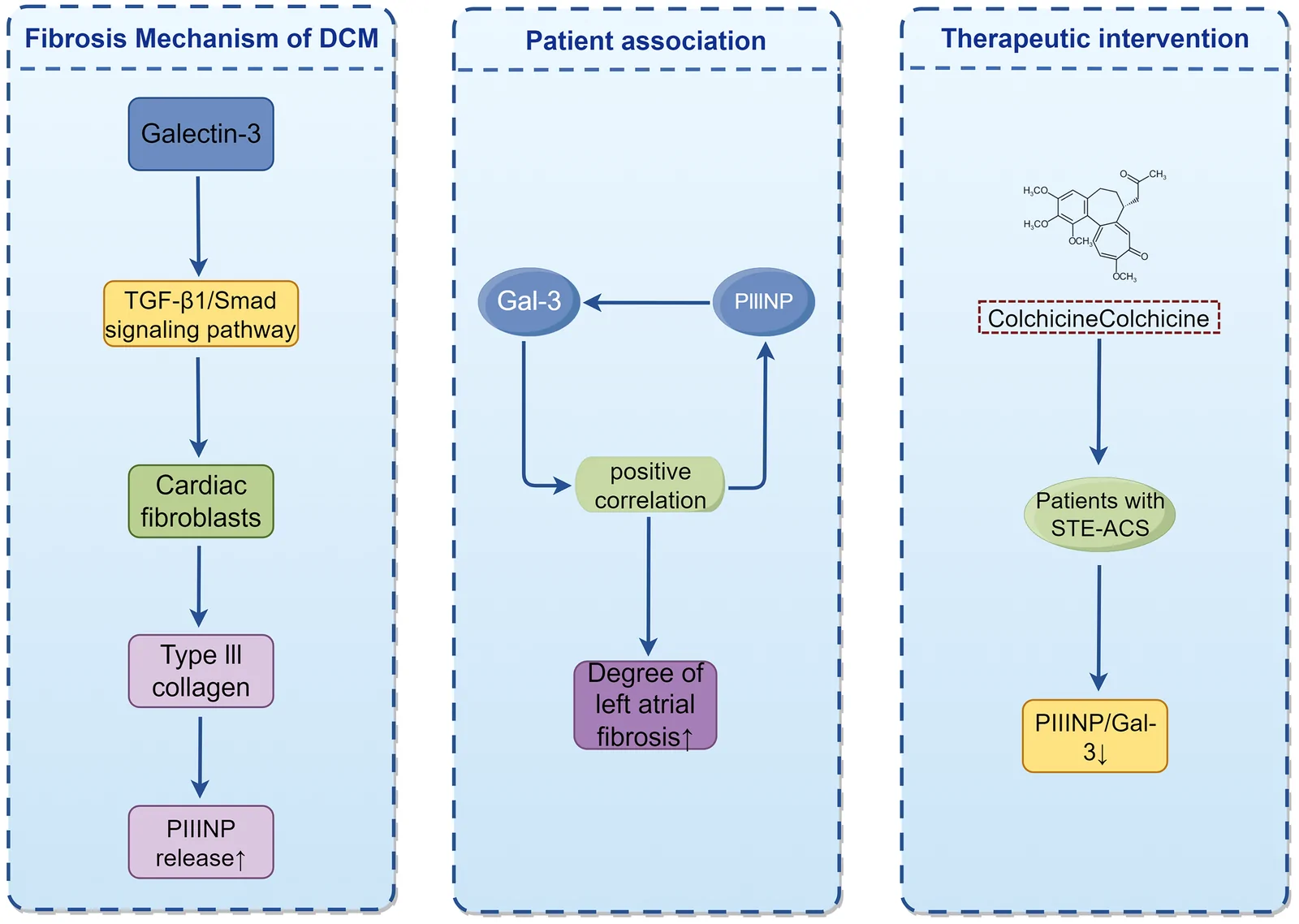
**In dilated cardiomyopathy (DCM), Galectin-3 (Gal-3) facilitates the release of more type III procollagen N-terminal propeptide (PIIINP) by activating the TGF-β1/Smad signaling pathway, which induces cardiac fibroblasts to generate abundant type III collagen**. Clinically, Gal-3 is positively correlated with PIIINP levels in patients suffering from metabolic syndrome and atrial fibrillation, and both factors synergistically accelerate fibrosis development. As for therapeutic application, colchicine can lower PIIINP concentrations and regulate Gal-3 expression among patients with acute coronary syndrome.

The combined assessment approach provides notable benefits for monitoring disease progression and evaluating treatment efficacy [32]. In patients with dilated cardiomyopathy, serial measurements of circulating Gal-3 have been linked to fibrosis markers like PIIICP and OPN. While circulating Gal-3 does not directly correlate with biopsy-determined fibrosis severity, it is strongly associated with adverse cardiovascular outcomes. In contrast, PIIINP, a direct product of collagen synthesis, may more accurately reflect extracellular matrix remodeling. In the Heart ‘OMics’ in AGing (HOMAGE) trial, which focused on high-risk heart failure patients, spironolactone treatment did not significantly alter PIIINP levels but did significantly reduce PICP levels, indicating decreased type I collagen synthesis. Additionally, the reduction in PICP was linked to improved diastolic function. These findings imply that a combined assessment of Gal-3 and PIIINP could provide a more comprehensive evaluation of anti-fibrotic treatment efficacy.

## 5. Predictive Value of Gal-3 and PIIINP for Major Adverse Cardiovascular Events

### 5.1 Clinical Evidence for Gal-3 as a Predictor of Cardiovascular Events

Elevated Gal-3 levels are closely associated with an increased risk of emergency hospitalization in patients with DCM and independently predict MACE. In DCM patients, Gal-3 levels positively correlate with PIIINP and OPN, suggesting Gal-3’s role in promoting myocardial fibrosis and disease progression. Specifically, DCM patients with baseline Gal-3 levels of ≥14.54 ng/mL have a 2.6-fold higher risk of emergency hospitalization, regardless of traditional risk factors like left ventricular ejection fraction. Furthermore, changes in Gal-3 levels over time have prognostic significance: newly diagnosed DCM patients showed significant decreases in Gal-3 levels at 3-month and 12-month follow-ups, with greater reductions in those without endpoint events. This indicates that Gal-3 levels may serve as indicators of treatment efficacy [3].

Extrapolated findings indicate that patients with acute myocardial infarction (AMI) who have Gal-3 levels above 9.2 ng/mL face a ninefold increased risk of reinfarction. This risk is further exacerbated by the presence of carotid plaque [33]. Another study focusing on ST-segment elevation myocardial infarction (STEMI) patients found that those with high Gal-3 levels experienced significantly higher rates of major cardiovascular events both during hospitalization and at 1-year follow-up [34]. This group also showed a strong association with an increased risk of developing heart failure and requiring rehospitalization [35]. Importantly, combining the assessment of Gal-3 with left ventricular ejection fraction (LVEF) enhances the ability to stratify risk [35].

### 5.2 Role of PIIINP in Predicting Cardiovascular Event Risk

Elevated serum levels of PIIINP are biomarkers indicating the severity of myocardial fibrosis and are closely associated with structural heart abnormalities. In patients with aortic stenosis (AS), although PIIINP does not directly correlate with diffuse fibrosis markers identified by CMR, its dynamic trend suggests it may serve as a surrogate indicator for fibrosis progression [36]. In individuals with DCM, PIIINP significantly correlates with declining left ventricular function, and elevated baseline levels predict the risk of emergency hospitalization within 12 months. Furthermore, in patients with preserved left ventricular ejection fraction after STEMI, a PIIINP level of ≥381.4 ng/mL on the 12th hospital day significantly predicts the risk of myocardial fibrosis at one year. These findings collectively highlight PIIINP’s potential as a prognostic assessment tool.

Extrapolated findings indicate that in aortic stenosis, PIIINP is a marker of fibrosis progression. In post-STEMI patients, PIIINP predicts late-stage fibrosis. When combined with cardiac function markers, PIIINP significantly enhances risk stratification accuracy. In patients with acute decompensated heart failure (ADHF), integrating PIIINP with NT-proBNP improves the predictive performance of clinical models. For individuals with atrial fibrillation, PIIINP is linked to alterations in gut microbiota and specific metabolites; its elevation may exacerbate fibrosis through the microbiome-metabolism axis. Thus, incorporating PIIINP into existing risk assessment frameworks not only aids in identifying high-risk patients but also supports biomarker-guided, individualized anti-fibrotic therapy.

### 5.3 Dynamic Monitoring of Gal-3 and PIIINP for Event Prediction and Application Prospects

Monitoring changes in Gal-3 and PIIINP is crucial for assessing disease progression, treatment efficacy, and predicting MACE in patients with DCM. In these patients, fluctuations in Gal-3 levels are closely linked to the risk of cardiovascular events. A study involving 70 newly diagnosed DCM patients, tracked over 12 months, revealed that those with baseline Gal-3 levels of 14.54 ng/mL or higher had a significantly increased risk of death or emergency hospitalization. Furthermore, serial measurements showed that Gal-3 levels were elevated at the onset in newly diagnosed patients but gradually decreased over time. Conversely, in chronic DCM patients, Gal-3 levels remained relatively stable, indicating that these patterns may reflect the progression of fibrosis related to the disease.

PIIINP, a marker of collagen synthesis, is also a predictive indicator due to its dynamic variations. In individuals with NIDCM, PIIINP levels are closely associated with the extent of LGE observed through CMR. Those with higher baseline PIIINP levels have a significantly increased risk of ventricular arrhythmias and hospitalization due to heart failure. Furthermore, in patients with type 2 diabetes and heart failure, elevated baseline PIIINP levels are strongly linked to the development of heart failure with preserved ejection fraction (HFpEF) within three years, highlighting its importance in ongoing monitoring.

Monitoring Gal-3 and PIIINP together enhances the predictive accuracy of cardiovascular events. In patients with NIDCM, combining Gal-3, PIIINP, and LGE significantly improves cardiovascular event prediction. Additionally, tracking changes in Gal-3 and PIIINP effectively assesses treatment efficacy. For instance, spironolactone therapy significantly reduces PICP levels, a marker of type I collagen synthesis, while having minimal impact on PIIINP, highlighting different responses among collagen markers. Thus, dynamic monitoring of Gal-3 and PIIINP not only helps evaluate myocardial fibrosis severity and disease progression in DCM patients but also provides crucial evidence for clinical treatment decisions and prognosis evaluation. These biomarkers significantly enhance risk prediction models for DCM, and their integration into prognostic systems substantially increases predictive accuracy.

## 6. Future Research Directions and Clinical Application Prospects

### 6.1 Deepening Mechanistic Research

Gal-3, a member of the β-galactoside-binding lectin family, is vital in regulating myocardial fibrosis in DCM through various molecular pathways [9]. The interaction between Gal-3 and PIIINP is essential for understanding the shift from inflammatory injury to ongoing ECM remodeling in DCM. In a rat model of acute myocardial infarction, Gal-3 expression was significantly linked to the activation of the Notch1-TGF-β-Smads pathway. Atorvastatin was shown to reduce Gal-3 and collagen expression by inhibiting this pathway, highlighting Gal-3’s crucial role in fibrosis. Furthermore, Gal-3 influences fibrosis progression through epigenetic mechanisms. Sirtuin 2 (SIRT2) mediates the deacetylation of histone H3 lysine 27 (H3K27) in the Gal-3 promoter region, inhibiting its transcription and reducing radiation-induced myocardial fibrosis. This regulatory mechanism underscores the epigenetic flexibility of Gal-3 expression, offering new insights for targeted therapeutic interventions.

Interactions between Gal-3 and ECM components play a crucial role in DCM. In DCM patients, serum levels of Gal-3 positively correlate with OPN, TIMP-1, and PIIICP. This correlation indicates that Gal-3 is involved in regulating ECM degradation and maintaining fibrosis balance [3]. The interaction between Gal-3 and PIIINP is particularly pronounced in non-ischemic DCM. Both markers are directly linked to LGE mass in cardiac MRI, which reflects the severity of myocardial replacement fibrosis. Further studies using ultrasound-targeted microbubble disruption technology to deliver Gal-3 shRNA showed a significant decrease in the I/III collagen ratio in myocardial infarction rats. This result confirms Gal-3’s regulatory role in collagen subtype distribution.

The interaction between Gal-3 and the TLR4/MyD88/NF-κB inflammatory pathway exacerbates fibrosis at the signaling level. Modified citrus pectin (MCP), a Gal-3 inhibitor, reduces the release of proinflammatory factors like IL-1β and TNF-α by downregulating this pathway, thus easing myocardial fibrosis. Additionally, Gal-3 influences the fibrotic microenvironment by affecting macrophage polarization. During myocardial infarction repair, Gal-3 promotes M2 macrophage polarization, increasing the expression of arginase-1 (Arg-1) and IL-10. Conversely, Gal-3C protein inhibitors disrupt this process and reduce fibrotic signaling cascades [11]. These insights provide a molecular understanding of Gal-3’s pivotal role in the fibrotic network (Fig. 4).

**Fig. 4.**
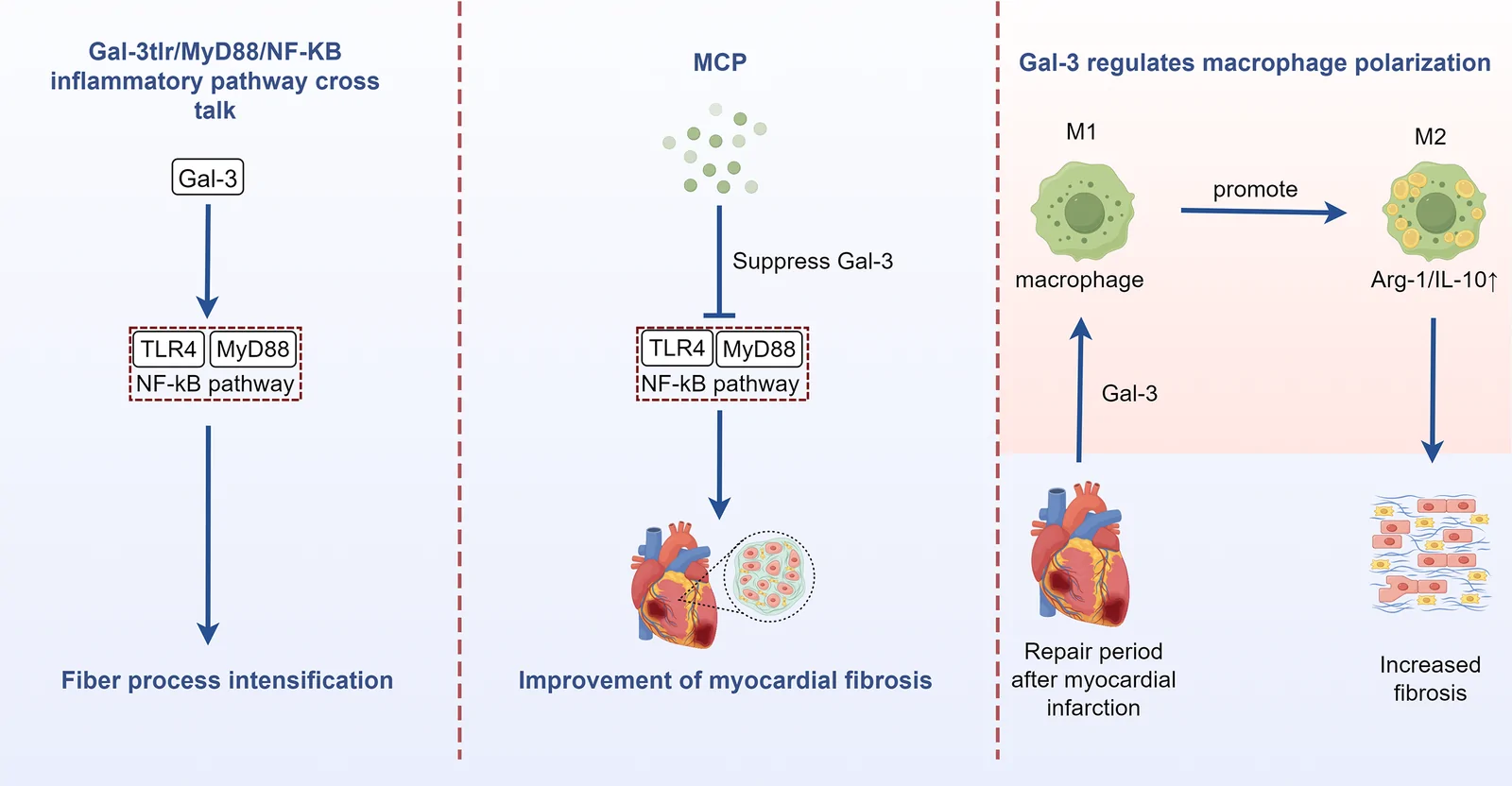
**Galectin-3 (Gal-3) exacerbates fibrosis via activating the Toll-like receptor (TLR)/myeloid differentiation primary response protein 88 (MyD88)/nuclear factor-kappa B (NF-κB) inflammatory pathway**. Modified pectin can alleviate myocardial fibrosis by suppressing Gal-3 activity. During the repair phase following myocardial infarction, Gal-3 mediates macrophage polarization toward the M2 phenotype, elevates the expression of arginase-1 and interleukin (IL)-10, and ultimately aggravates fibrotic lesions.

In summary, Gal-3 facilitates myocardial fibrosis by orchestrating inflammation, ECM metabolism, and epigenetic regulation [9]. It interacts with ECM markers such as PIIINP, forming a dynamic regulatory network. Future research should focus on elucidating the time-dependent roles of Gal-3 during different fibrosis stages and exploring its potential as a multi-target therapeutic hub in clinical settings [37].

Unified Mechanistic Model of Gal-3-Driven Myocardial Fibrosis in DCM: Gal-3 functions as a key upstream regulator, integrating several pathways in the development of myocardial fibrosis in DCM. (1) Hippo Pathway: In the early stages of DCM, activation of the Hippo pathway leads to increased Gal-3 expression [38]. (2) NF-κB Pathway: Gal-3 activates the TLR4/MyD88/NF-κB pathway, which promotes inflammation and macrophage polarization. (3) TGF-β/Smad Pathway: Gal-3 stabilizes TGF-β receptors, enhancing Smad2/3 phosphorylation and activating fibroblasts [10]. (4) SIRT Pathway: Gal-3 suppresses SIRT2/7, affecting epigenetic regulation and maintaining its own expression. Collectively, these pathways contribute to collagen synthesis (indicated by elevated PIIINP/PICP levels), extracellular matrix remodeling, and the progression of myocardial fibrosis in DCM.

### 6.2 Development of Novel Detection Technologies

As research into myocardial fibrosis mechanisms in DCM advances, Gal-3 and PIIINP have become key biomarkers. Developing precise detection methods is crucial for early diagnosis and ongoing monitoring. Studies indicate that serum Gal-3 levels in DCM patients significantly correlate with adverse cardiovascular outcomes. An optimal cutoff of 14.54 ng/mL has been identified, with patients above this level facing a 2.6-fold increased risk of events. This finding emphasizes the need for standardized Gal-3 detection methods for clinical risk stratification [39]. Additionally, PIIINP, a direct marker of collagen synthesis, shows a strong correlation with the severity of LGE in NIDCM patients, underscoring its diagnostic value in assessing myocardial fibrosis.

Current assay development primarily focuses on enhancing analytical performance. For Gal-3, the main challenge is to clarify its unclear direct correlation with fibrosis. Achieving this requires increased specificity, which can be attained through optimized antibody selection and refined detection conditions. In the case of PIIINP detection, its short half-life and high variability call for the development of more stable assay methods. Additionally, dynamic monitoring technologies are becoming increasingly important, as Gal-3 exhibits time-dependent characteristics in newly diagnosed DCM patients. This scenario necessitates detection methods that provide exceptional repeatability and precision.

Future directions in diagnostic technology development should prioritize several key areas. First, establishing standardized protocols for sample collection and processing is crucial. Second, developing automated testing platforms can significantly streamline operations. Third, exploring novel detection technologies will enhance both sensitivity and specificity. Fourth, integrating artificial intelligence algorithms can optimize biomarker combinations and threshold determinations. These advancements will enable early identification and precise monitoring of myocardial fibrosis in DCM, providing more reliable evidence for clinical decision-making.

### 6.3 Exploration of Personalized Treatment Strategies

In recent years, personalized therapies targeting Gal-3 and PIIINP have shown significant promise in treating myocardial fibrosis in DCM. Evidence indicates that Gal-3 levels of 14.54 ng/mL or higher and PIIINP levels of 97 ng/mL or higher are linked to poor prognosis in DCM patients, as confirmed by multiple clinical studies. These biomarkers correlate with the severity of fibrosis and independently predict adverse cardiovascular events, supporting their use in risk stratification. Some proposals suggest that dynamic monitoring of these biomarkers could guide personalized anti-fibrotic therapy.

Therapies targeting the inflammation-fibrosis axis could benefit patients with persistently high Gal-3 levels [40]. In models of chronic Chagas cardiomyopathy, inhibiting STAT3 improved fibrosis, even though Gal-3 levels remained unchanged. Evidence indicates that renin-angiotensin system inhibitors can reduce myocardial extracellular volume, thereby supporting collagen modulation [41]. Speculatively, interventions focusing on collagen metabolism, such as early use of ACE inhibitors, might be advantageous for patients with elevated PIIINP.

Research indicates that elevated levels of both Gal-3 and PIIINP are more strongly associated with an increased risk of adverse events than either marker alone. In newly diagnosed DCM patients, Gal-3 levels typically decrease over 3 to 12 months, whereas in chronic patients, these levels tend to remain stable. Although not yet supported by evidence, it is suggested that Gal-3 and PIIINP be monitored every 3 to 6 months in conjunction with standard therapy. If both markers remain elevated, intensified anti-fibrotic treatment should be considered.

### 6.4 Multicenter, Large-Sample Clinical Studies

Multicenter, large-scale clinical studies are crucial for validating the predictive value and clinical utility of biomarkers across diverse populations [42]. In the context of DCM, research has increasingly focused on Gal-3 and PIIINP due to their association with myocardial fibrosis and clinical outcomes. Studies have shown that Gal-PIIINP levels of 3 levels correlate with fibrosis markers such as OPN, TIMP-1, PIIICP, and MMP-2 [10]. However, Gal-3 levels do not correlate with fibrosis severity as determined by endomyocardial biopsy. These findings indicate that although Gal-3 does not directly correlate with histological fibrosis, its dynamic changes reflect fibrotic activity and have significant predictive value for cardiovascular outcomes in DCM patients.

A study confirmed the predictive power of Gal-3 and PIIINP in patients with NIDCM. Fibrosis assessment using LGE on CMR revealed that patients with LGE (LGE+) had significantly higher serum levels of Gal-3, PICP, and PIIINP. These biomarker levels were directly correlated with LGE mass. Kaplan-Meier analysis indicated that Gal-3 levels above 13.8 ng/dL and PICP levels over 97 ng/dL significantly predicted the risk of ventricular arrhythmia and heart failure hospitalization. Combining LGE with Gal-3 and PICP markedly improved the prediction of outcomes in NIDCM patients. These multicenter studies collectively demonstrate that collagen metabolism markers, such as Gal-3 and PIIINP, are closely linked with myocardial fibrosis as assessed by CMR. They effectively predict major adverse cardiovascular events in DCM patients, providing robust biomarker tools for clinical risk stratification and prognostic assessment.

### 6.5 Combined Biomarker and Imaging Assessment

Recent advancements in CMR have positioned LGE as a leading non-invasive method for detecting myocardial replacement fibrosis. Despite this, endomyocardial biopsy remains the histological reference standard. However, relying solely on imaging may not fully capture the condition’s dynamic progression. By integrating biomarkers with CMR, we can enhance diagnostic and risk assessment capabilities for myocardial fibrosis in patients with NIDCM. Research indicates that markers of collagen metabolism, such as Gal-3, PICP, and PIIINP, show significant correlation with LGE positivity and LGE mass. Notably, when Gal-3 levels exceed 13.8 ng/dL and PICP levels surpass 97 ng/dL, these markers effectively predict major adverse cardiovascular events. These collagen metabolism markers are also closely tied to the severity of heart failure. In patients with a left ventricular ejection fraction below 30%, they offer predictive value beyond LGE alone. Combining LGE with biomarkers like Gal-3 and PICP significantly improves prognostic predictions in NIDCM patients, aiding in the development of personalized treatment strategies. To support clinical application, we propose a risk stratification algorithm that integrates Gal-3, PIIINP, and CMR-LGE: (1) **Low risk**: Gal-3 <14.54 ng/mL, PIIINP <97 ng/dL, and LGE-negative. Annual MACE risk is <5%. Recommend standard heart failure therapy and biomarker monitoring every 6–12 months. (2) **Intermediate risk**: Either Gal-3 ≥14.54 ng/mL or PIIINP ≥97 ng/dL, or LGE-positive (single lesion). Annual MACE risk is 5–15%. Intensify anti-fibrotic therapy and monitor biomarkers every 3–6 months. (3) **High risk**: Gal-3 ≥14.54 ng/mL and PIIINP ≥97 ng/dL, or multi-site LGE. Annual MACE risk is >15%. Consider advanced heart failure management and close surveillance. Future research should investigate various biomarker combinations and multimodal imaging assessments to achieve precise monitoring and intervention for myocardial fibrosis.

### 6.6 Mechanisms of Fibrosis Reversal and Regenerative Medicine

Reversing myocardial fibrosis and exploring regenerative strategies offer promising avenues for treating DCM, with a focus on Gal-3 and PIIINP-guided antifibrotic therapy. Collagen turnover markers, PICP and PIIINP, are indicative of fibrosis reversal. Spironolactone, for instance, reduces PICP levels and enhances diastolic function. However, changes in PIIINP and Gal-3 are less consistent, suggesting varied responses among these markers. Emerging regenerative approaches aim to complement antifibrotic treatments, with Gal-3 being a crucial therapeutic target. Inhibiting Gal-3 not only reduces fibrosis but also fosters a pro-regenerative microenvironment. Further research is necessary to validate the effectiveness of combined Gal-3/PIIINP-guided therapy alongside regenerative strategies.

## 7. Conclusion

Gal-3 plays a vital role in regulating myocardial fibrosis and is well-known for its pathophysiological functions. Unlike markers that indicate static fibrosis, Gal-3 reflects ongoing fibrosis activity. Its significance stems from its dual role in mediating inflammatory responses and remodeling the ECM. By activating fibroblasts and encouraging collagen deposition, Gal-3 contributes to the progression of myocardial fibrosis, offering new perspectives on the pathogenesis of dilated cardiomyopathy. However, studies show inconsistent correlations between circulating Gal-3 levels and fibrosis, suggesting that this biomarker may better represent active fibrosis rather than permanent structural changes.

In clinical settings, PIIINP often complements Gal-3, though not universally. PIIINP acts as a non-specific marker of systemic collagen synthesis, indicating collagen turnover in various organs, including the heart. PICP and PIIICP enhance its detection, making the combined measurement of Gal-3, PIIINP, PICP, and PIIICP an effective strategy for assessing changes in myocardial fibrosis while considering systemic confounding factors. From a translational medicine perspective, this multi-marker approach not only enhances diagnostic sensitivity but also distinguishes different pathophysiological stages of fibrosis, providing valuable biological insights for clinical decision-making.

In prognostic evaluations, Gal-3 is widely recognized for its predictive value, as supported by numerous studies. However, variability in detection methods hinders comparability between these studies. Gal-3’s predictive capability likely stems from its association with fibrosis and myocardial remodeling processes. This broad-spectrum attribute makes it a valuable tool for risk stratification in dilated cardiomyopathy. Despite its potential, caution is necessary due to variations in detection methods and inconsistent cutoff values, which may compromise result comparability. Future research should prioritize developing standardized detection protocols to resolve these issues.

Looking ahead, three key areas warrant attention. First, understanding the molecular mechanisms of Gal-3 in myocardial fibrosis is crucial, particularly its interactions with other fibrosis-related pathways. Second, optimizing detection technologies is vital, which involves developing highly sensitive assays and establishing standardized cutoff values. Finally, validating personalized strategies is necessary; while current biomarker cutoffs are based on evidence, monitoring intervals and treatment algorithms are still speculative and require large-scale trials. These advancements will transition biomarkers from research tools to routine clinical applications, ultimately improving diagnosis and treatment outcomes for patients with dilated cardiomyopathy.
